# Purification and Biological Properties of Raniseptins-3 and -6, Two Antimicrobial Peptides from *Boana raniceps* (Cope, 1862) Skin Secretion

**DOI:** 10.3390/biom13030576

**Published:** 2023-03-22

**Authors:** Gabriel Gonçalves de Freitas, João Martins Barbosa, Carlos José Correia de Santana, Ana Carolina Martins Magalhães, Keven Wender Rodrigues Macedo, Jéssica Oliveira de Souza, Jessica Schneider de Castro, Isadora Alves de Vasconcelos, Amanda Araújo Souza, Sonia Maria de Freitas, Sônia Nair Báo, Samuel Ribeiro Costa, Guilherme Dotto Brand, Ian de Meira Chaves, Vivian Vasconcelos Costa, Wagner Fontes, Osmindo Rodrigues Pires Júnior, Mariana S. Castro

**Affiliations:** 1 Laboratory of Toxinology, Department of Physiological Sciences, Institute of Biological Sciences, University of Brasilia, Brasilia 70.910-900, DF, Brazil; 2 Laboratory of Protein Chemistry and Biochemistry, Department of Cell Biology, Institute of Biological Sciences, University of Brasilia, Brasilia 70.910-900, DF, Brazil; 3 Brazilian Biosciences National Laboratory (LNBio), National Center for Research in Energy and Materials (CNPEM), Campinas 13083-970, SP, Brazil; 4 Laboratory of Biophysics, Department of Cell Biology, Institute of Biological Sciences, University of Brasilia, Brasilia 70.910-900, DF, Brazil; 5 Electron Microscopy Laboratory, Department of Cell Biology, Institute of Biological Sciences, University of Brasilia, Brasilia 70.910-900, DF, Brazil; 6 Laboratory of Synthesis and Analysis of Biomolecules, Institute of Chemistry, University of Brasilia, Brasilia 70.910-900, DF, Brazil; 7 Center for Research and Development of Pharmaceuticals, Department of Morphology, Institute of Biological Sciences, Federal University of Minas Gerais, Belo Horizonte 31270-901, MG, Brazil

**Keywords:** anurans, *Boana raniceps*, skin secretion, antimicrobial peptides, Raniseptins

## Abstract

The number of multidrug-resistant pathogenic microorganisms has been growing in recent years, most of which is due to the inappropriate use of the commercial antibiotics that are currently available. The dissemination of antimicrobial resistance represents a serious global public health problem. Thus, it is necessary to search for and develop new drugs that can act as antimicrobial agents. Antimicrobial peptides are a promising alternative for the development of new therapeutic drugs. Anurans’ skin glands are a rich source of broad-spectrum antimicrobial compounds and hylids, a large and diverse family of tree frogs, are known as an important source of antimicrobial peptides. In the present study, two novel antimicrobial peptides, named Raniseptins-3 and -6, were isolated from *Boana raniceps* skin secretion and their structural and biological properties were evaluated. Raniseptins-3 and -6 are cationic, rich in hydrophobic residues, and adopt an α-helix conformation in the presence of SDS (35 mM). Both peptides are active against Gram-negative bacteria and Gram-positive pathogens, with low hemolytic activity at therapeutic concentrations. No activity was observed for yeasts, but the peptides are highly cytotoxic against B16F10 murine melanoma cells and NIH3T3 mouse fibroblast cells. None of the tested compounds showed improvement trends in the MTT and LDH parameters of MHV-3 infected cells at the concentrations tested.

## 1. Introduction

Antimicrobial resistance (AMR) is an ancient natural phenomenon related to the evolutionary biology of bacteria. Unfortunately, the misuse and long-term use of conventional antibiotics have led to an acceleration in the resistance process, causing a serious public health problem at a global level [1,2,3,4,5]. The World Health Organization (WHO) considers infections caused by multidrug-resistant bacteria to be one of the greatest threats to human health of the 21st century [2,6], and this problem is perceptible when faced with infections that were hitherto curable and are currently refractory, such as methicillin-resistant *Staphylococcus aureus* (MRSA) infection [7].

The rapid spread of AMR has driven many efforts by researchers to seek new compounds and antimicrobial strategies to limit this phenomenon and ensure the treatment of microbial infections. Faced with these challenges, anurans have proved to be an impressive source of biologically active compounds, mainly peptides with antimicrobial activity, with potential for the development of new drugs aimed at the treatment of infections caused by multidrug-resistant microorganisms [8,9,10,11,12].

Antimicrobial peptides (AMPs) are molecules that can vary in size, sequence, net charge, hydrophobicity, three-dimensional structure and spectrum of action. They are small molecules (<100 amino acid residues), positively charged (between +2 and +6 at pH 7.0) and about 50% of the amino acid residues present in their peptide chains are hydrophobic, and amphipathic (have both hydrophobic and hydrophilic domains) [13,14].

AMPs, in general, act by producing disturbances in the bacterial membrane through electrostatic and hydrophobic interactions, leading to the formation of pores and destabilization of biological membranes. These electrostatic interactions occur between cationic residues and the electronegative surface of bacterial phospholipids such as phosphatidylglycerol, phosphatidylserine, cardiolipin and other types of phospholipids, such as lipopolysaccharides (LPS) for Gram-negative bacteria, and teichoic and teichuronic acids for Gram-positive bacteria [14]. In general, this disturbance causes the formation of pores or micellization of the bacterial membrane [15]. These peptides are capable of inducing their activities directly through interaction with the biological membrane of the pathogens or indirectly modulating host immunity through the recruitment/activation of immunocytes or by mediating Toll-like receptor (TLR) recognition of microbial products and nucleic acids released with tissue damage [16].

**Figure 1:**
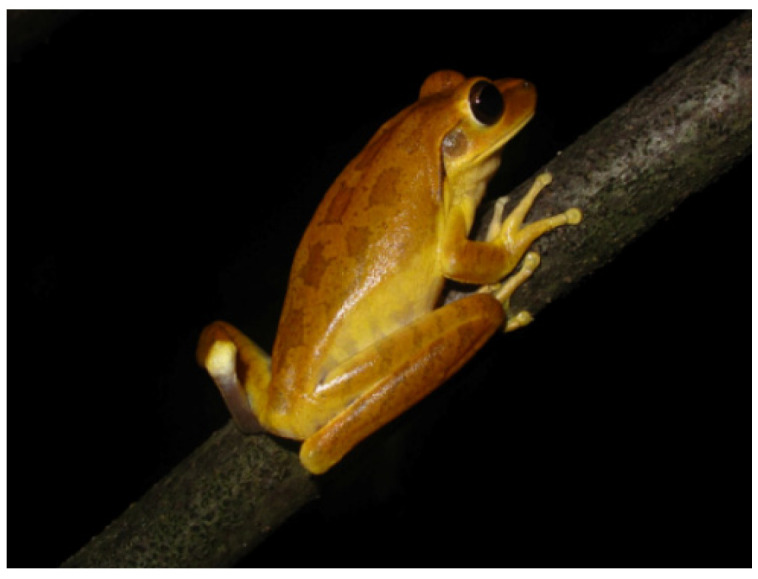
Adult specimen of *Boana raniceps*. Photo courtesy by Prof. Dr. Pedro Ivo Simões (Pernambuco Federal University, Recife, Brazil).

Hylids, a large and diverse family of tree frogs, are known as an important source of antimicrobial peptides, with highly conserved features [17,18]. *Boana raniceps* (Cope, 1862), the target species of the present study, is a neotropical hylid of large size with a snout–vent length ranging from 70 to 75 mm. It has a slender body and limbs, broad head, smooth back with a yellowish cream color and uniformly colored belly. The presence of transverse bands on their back is common [19] (Figure 1). These animals can be commonly found in the Brazilian Cerrado and Caatinga, and their distribution ranges from Amazonian Colombia (vicinity of Leticia) and Venezuela (Amazonas) to French Guiana, Brazil (central Amazonia to Bahia), Paraguay, northern Argentina, and eastern Bolivia [20]. Its population is considered to be stable and can be found in degraded habitats and urban areas [21]. This species is associated with rivers or large swamps [22].

The aim of the present study was to carry out peptidomic analysis of the skin secretion of the hylid *B. raniceps*, leading to the purification and characterization of the biological properties of two novel Raniseptins.

## 2. Materials and Methods

### 2.1. Collection of Skin Secretion

The adult specimens were collected by means of an active search during the night. The collections were carried out in the municipality of Monte Alegre de Goiás, in the state of Goiás, Brazil, under the SISBIO/ICMBio license number 75407-1, and under the SISGEN protocol number AB8B1C1. The crude secretion was obtained with the aid of moderate electrical stimulation of a direct current of 50 V with low amperage, 500 mA. The released secretion was collected by washing the animal’s body with Milli-Q water, then frozen, lyophilized, and stored at −20 °C until the time of use. The skin-secretion-harvesting procedure was approved by the Animal Ethics Committee of the University of Brasilia.

### 2.2. Fractionation of Crude Secretion by Reverse-Phase High-Performance Liquid Chromatography (RP-HPLC)

Aliquots of the freeze-dried *Boana raniceps* crude secretion were prepared in 10 mg/mL of water with 0.12% (*v*/*v*) trifluoroacetic acid (TFA), homogenized and then centrifuged at 13,000 rpm for 5 min. Then, 100 µL of the supernatant was injected into a C_18_ column (Shim-pack VP-ODS, no. 2122095, 4.6 mm × 150 mm, 5 µm). Elution was carried out at a flow of 1 mL/min with a gradient of acetonitrile with 0.12% (*v*/*v*) TFA. Elution was monitored at 216 and 280 nm and fractions were collected, dried and stored at −20 °C.

### 2.3. Purification of Antimicrobial Peptides

The fraction of interest was rechromatographed to purify the antimicrobial peptides. Dried aliquots were prepared in water with 0.12% (*v*/*v*) TFA, homogenized and centrifuged at 13,000 rpm for 5 min. The supernatant was injected into a normal phase hydrophilic interaction liquid chromatography column (HILIC) (Phenomenex Luna 200A, no. H18-129976, 4.6 mm × 250 mm, 5 µm). Chromatography was performed on a linear gradient of 10–30% acetonitrile with 0.12% (*v*/*v*) TFA, at a flow rate of 1 mL/min and monitored at 216 and 280 nm.

### 2.4. Mass Spectrometry

The fractions of interest were analyzed by matrix-assisted laser desorption ionization time of flight mass spectrometry (MALDI-TOF MS) using an AutoFlex II equipment (Bruker, Germany), in positive ion reflectron mode with *m*/*z* range of 700–4000. The matrix used for ionization was α-cyano-4-hydroxycinnamic acid and the calibration was performed using the Peptide Calibration Standard II (Bruker, Germany), composed of a mixture of Bradykinin (757.399 Da), Angiotensin I (1046.5418 Da), Angiotensin II (1296.6848 Da), Substance P (1347.7354 Da), Bombesin (1619.8223 Da), ACTH_clip (1–17) (2093.0862 Da), ACTH_clip (18–39) (2465.1983 Da) and Somatostatin (3147.4710 Da).

### 2.5. Chemical Sequencing

The isolated peptides were sequenced by Edman degradation, using an automatic peptide and protein sequencer (model PSSQ 33A, Shimadzu, Japan), which was previously calibrated with a standard phenylthiohydantoin (PTH) amino acid mixture.

### 2.6. Circular Dichroism

The structural analysis of the peptides was performed using circular dichroism measurements on the spectropolarimeter model J-815 equipped with a Peltier-type temperature control system, coupled to a water pump (Jasco, Japan). The samples were analyzed in a 0.05 cm quartz cuvette, in the presence of Milli-Q water and, as a membrane mimic, 35 mM of sodium dodecyl sulfate (SDS) at 25 °C was used. Each peptide spectrum in the Far-UV range (190–260 nm) was obtained from 10 consecutive measurements and after subtraction of the corresponding water and SDS spectra. The observed ellipticity was converted into molar ellipticity ([*θ*]) (degree·cm^2^·dmol^−1^) based on the molecular mass per residue of 112 Da. The α-helix secondary structure content was estimated considering the values of molar ellipticity (degree·cm^2^·dmol^−1^) in λ_208nm_ based on [23].

### 2.7. Bioinformatic Analysis

The BLAST software (https://blast.ncbi.nlm.nih.gov, accessed on 20 October 2022) [24] was used to identify similar peptide sequences; the APD3 database (the Antimicrobial Peptide Database, http://aps.unmc.edu/AP/main.php, accessed on 20 October 2022) [25] was also used, with the purpose of determining the degree of identity or similarity with the peptides available in the database; Emboss Needle (https://www.ebi.ac.uk/Tools/psa/emboss_needle/) [26] was used for the alignment of peptide sequences; NetWheels (http://tools.alanmol.com.br/NetWheels/) [27], SOPMA software (https://npsa-prabi.ibcp.fr) [28], I-Tasser (https://zhanglab.ccmb.med.umich.edu/I-TASSER/) and several tools available at https://www.expasy.org/ [29] were used to obtain the physicochemical properties of the peptides, helical wheel projections, secondary structure prediction and three-dimensional structure model prediction.

### 2.8. Solid-Phase Synthesis and Purification

The Raniseptin-3 and -6 peptides were synthesized by the solid-phase method and purified as previously described by [30,31]. Fmoc-Gln(trt)-Wang resin (Sigma-Aldrich) was used. The qualitative effectiveness of each step was evaluated by the Kaiser test, and the peptide cleavage step was performed with TFA, triisopropylsilane (TIS), and H_2_O in the proportion of 95/2.5/2.5, *v*/*v*. In the final step, the resin was solubilized for 1 h and 30 min, and then the samples were evaporated in argon, as described by [30]. The synthetic peptides were purified by RP-HPLC using an analytical C_18_ reversed-phase column (Shim-pack VP-ODS, no. 2122095, 4.6 mm × 150 mm, 5 µm), and the molecular masses of both peptides were confirmed by MALDI-TOF mass spectrometry.

### 2.9. Quantification of Peptides

The peptides were quantified according to the methodology described by [32]. A spectrophotometer (UV-M51, Bel Photonics, Brazil) was used for determining the UV absorption at 205, 215 and 225 nm.

### 2.10. Antibacterial Activity

The evaluation of the antibacterial activity was performed with the following Gram-negative and Gram-positive bacteria: *Escherichia coli* (ATCC 25922), *Klebsiella pneumoniae* (ATCC 13883), *Klebsiella pneumoniae* carbapenemase CAPB053, *Staphylococcus epidermidis* (ATCC 12228), and *Staphylococcus aureus* (ATCC 25923). Aliquots of 200 µL of the selected strains were incubated with 6.8 mL in Mueller–Hinton broth for 24 h at 37 °C. After the incubation period, the optical density (OD) at 625 nm was determined. Then, the bacterial suspensions were diluted and adjusted to an OD of 0.08–0.1, according to the CLSI (M7-A6) protocol [33]. This inoculum was diluted in the proportion of 1:200, for all the tested bacteria. Minimal inhibitory concentrations (MICs) were evaluated using 50 µL aliquots of each peptide (initial concentration: 256 µM) that were previously filtered through 0.22 µm filters (Millex GV, Millipore, Merck). Serial dilution was performed and incubation with 50 µL of the bacterial inoculum was carried out for 24 h at 37 °C. At the end of the incubation time, bacterial growth was evaluated at 620 nm using a Multiskan FC microplate reader (Thermo Scientific, San Jose, CA, USA).

The MIC was defined as the lowest concentration of peptide or other antimicrobial agents at which no growth was detectable after incubation at 37 °C for 24 h.

### 2.11. Antifungical Activity

The evaluation of the antifungal activity was performed with the yeast *Candida albicans* (ATCC 14053). Aliquots of 200 μL of the strain were resuspended in 6.8 mL of Brain Heart Infusion (BHI) broth, and then incubated under constant agitation for 24 h at 37 °C. To perform the test, an OD of 0.08–0.1 at 620 nm was obtained according to CLSI (M27-A3) [34], following by dilution in the proportion of 1:2000 in BHI broth. Minimal inhibitory concentrations (MICs) were evaluated using 50 µL aliquots of each peptide (initial concentration: 256 µM) that were previously filtered through a 0.22 µm filter (Millex GV, Millipore, Merck), and then diluted in series. Aliquots of 50 µL of the fungi suspension with approximately 5 × 10^2^ CFU/mL were applied to a 96-well plate and incubated for 24 h at 37 °C. The fungal growth was detected by reading the OD at 620 nm using a Multiskan FC microplate reader (Thermo Scientific, San Jose, CA, USA).

The MIC was defined as the lowest concentration of peptide or other antimicrobial agents at which no growth was detectable after incubation at 37 °C for 24 h.

### 2.12. Antiproliferative Activity

The antiproliferative activity was evaluated with the murine cutaneous melanoma cancer cell line B16F10 (ATCC CRL-6475) and mouse embryonic fibroblast NIH3T3 (ATCC CRL-1658). Cells were maintained as described by [35]. B16F10 and NIH3T3 cells were seeded in 75 cm^2^ culture flasks with complete medium (Dulbecco’s Modified Eagle’s Medium supplemented with 10% of fetal bovine serum, 100 IU/mL of penicillin and 100 µg/mL of streptomycin) and kept in an incubator at 37 °C in 5% CO_2_. After the incubation period, cells were seeded at a density of 5 × 10^3^/well in 96-well plates with complete medium and incubated overnight. Antiproliferative activity was evaluated using 50 µL aliquots of each peptide (initial concentration: 256 µM) that were previously filtered through a 0.22 µm filter (Millex GV, Millipore, Merck), and then serially diluted. After the treatment period, 15 µL aliquots of MTT (5 mg/mL in PBS, pH 7.4) with 135 µL of complete medium were added to the wells and incubated for 3 h. Formazan crystals were solubilized in 100 µL of DMSO, and then read at 595 nm on a Multiskan FC microplate reader (Thermo Scientific, San Jose-CA, USA).

### 2.13. Human Hemolysis Activity

Fresh human erythrocytes (O positive) were centrifuged and washed to remove plasma with 10 mM Tris-HCl buffer at pH 7.4, containing 150 mM NaCl (four washes, 2000 rpm for 3 min). To carry out the assay, a human erythrocytes suspension at 1% (*v*/*v*) was prepared, using the saline-Tris buffer.

Each peptide was resuspended in 100 µL of saline-Tris buffer (initial concentration: 256 µM) and then serially diluted. Then, aliquots of 100 µL of the 1% erythrocytes suspension were added and incubated for 1 h at room temperature. After the incubation time, the samples were centrifuged at 2000× *g* for 3 min and 100 µL aliquots of the supernatant were transferred to 96-well flat-bottom plates. The absorbance measurements were performed at 405 nm using a Multiskan FC plate reader (Thermo Scientific, San Jose, CA, USA).

For positive and negative controls, 1% (*v*/*v*) Triton-X and saline-Tris buffer were used. All tests were performed in triplicates and expressed as mean ± SD. The percentage of hemolysis was calculated based on [36].

### 2.14. Scanning Electron Microscopy (SEM)

Scanning electron microscopy was used to visualize the morphological alterations of *Escherichia coli* (ATCC 25922) induced by the incubation with Raniseptins-3 and -6. After the mid-log period, the bacteria were centrifuged at 13,000 rpm for 10 min, washed with 10 mM PBS (pH 7.6) and resuspended with an OD = 0.3, and then incubated with the peptides for 1 h and 30 min, using concentrations that corresponded to the MIC value. At the end of the incubation time, the cells were washed 3 times with PBS and centrifuged at 13,000 rpm for 5 min. Then, the pellets were fixed overnight with 0.1% Karnovsky, washed with buffer and dehydrated in a gradual series of acetone. After drying at the critical point and metallization of the material, the samples were observed using a JEOL JSM-7001F scanning electron microscope (Jeol Ltd., Akishima, Tokyo, Japan).

### 2.15. Infection and Viability Tests Protocol

The MHV-3 strain was provided and sequenced (GenBank accession no. MW620427.1) by [37]. L929 cells were distributed in a 96-well microtiter plate (1 × 10^5^ cells/well; 100 μL) and incubated at 37 °C in an atmosphere with 5% CO_2_, for 24 h. The medium was removed, and cells were inoculated with 100 μL of MHV-3 MOI = 0.01 and incubated for 16 h at 37 °C with 5% CO_2_. After incubation, viability tests were performed (MTT and LDH). Four different concentrations of the compound were tested. The following controls were used: a cell control (with DMEM 1% FBS); a cell control with DMEM 1% FBS plus the highest concentration of the tested compound; a virus control with MHV-3 inoculum and RPMI 1% FBS. Tests were performed on the technical duplicates.

After incubation, 10 μL of the MTT solution (5 mg/mL in PBS; final concentration of 0.2 μg/mL) was added to each well containing 90 µL of the supernatant. The plate was incubated at 37 °C with 5% CO_2_, for 120 min. After incubation, the entire content of the wells was discarded and the salt formed by the MTT reaction was dissolved with the addition of 100 µL of DMSO to each well, followed by constant agitation of the plate for 3 min at room temperature. The OD measurements were taken in a microplate spectrophotometer at 490 nm. With the absorbance values, we calculated the cell viability through the following equation: %cell viability = [(A × 100)/B], considering A as the sample to be compared to the control and B being the corresponding cell control.

For the LDH test, using LDH UV (Ref K084 Quibasa^®^), 4 µL of supernatant from each well was transferred to a new 96-well plate. 200 µL of the LDH kit’s working solution was added and 3 different readings were performed in a microplate spectrophotometer at 340 nm, with an interval of 1 min between each reading to calculate substrate consumption per minute. Values were expressed in absorbance (Δ/min).

Cell viability was calculated using the following equation:

Δ/min = [(Abs. 1st read − Abs. 2nd read) + (Abs. 2nd read − Abs. 3rd read) + (Abs. 3rd read − Abs. 4th read)]/3

## 3. Results

### 3.1. Purification of Antimicrobial Peptides from B. raniceps

**Figure 2:**
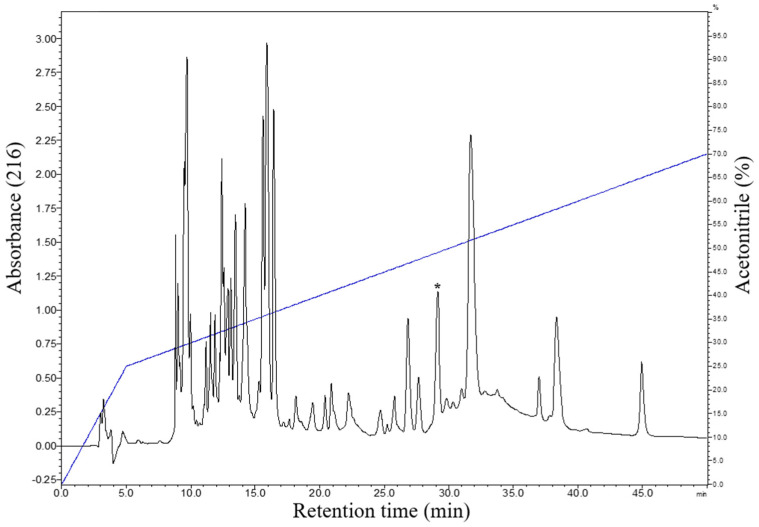
Typical chromatographic profile of the fractionation of the crude secretion of *B. raniceps*, using a C_18_ column (Shim-pack VP-ODS, no. 2122095, 4.6 mm × 150 mm, 5 µm) with a flow rate of 1 mL/min and detection at 216 nm. The fraction with antimicrobial activity that was characterized in the present study is indicated by an asterisk.

The cutaneous secretion obtained by electrical stimulation was lyophilized and subjected to fractionation by RP-HPLC using a C_18_ reverse-phase column. The chromatographic profile obtained with the fractionation of the cutaneous secretion of *B. raniceps* resulted in the elution of several chromatographic fractions (Figure 2). To identify the active fractions, an antimicrobial screening assay was performed using *E. coli* ATCC 25922 and *S. aureus* ATCC 25923, resulting in the identification of six fractions with antimicrobial activity.

**Figure 3:**
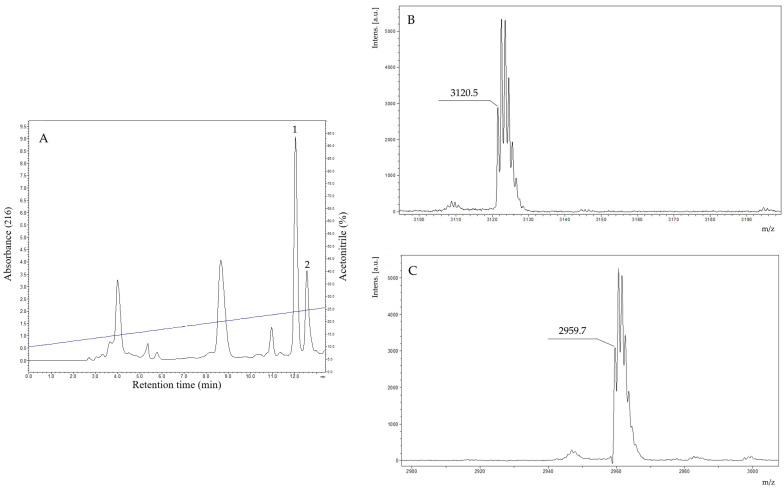
(**A**). Rechromatographic profile of the purification of the antimicrobial fraction found in the crude secretion of *B. raniceps*, using an HILIC column (Phenomenex Luna 200A, no. H18-129976, 4.6 mm × 250 mm, 5 µm) with a flow of 1 mL/min and detection at 216 nm. The fractions 1 and 2 (obtained after a rechromatographic step) were analyzed by MALDI-TOF MS and resulted in two purified peptides. (**B**). MALDI-TOF mass spectrum of fraction 1 (indicated in Figure 3A) exhibits a major component with a protonated molecular mass [M+H]^+^ of 3120.5 Da. (**C**). MALDI-TOF mass spectrum of fraction 2 (indicated in Figure 3A) exhibits a major component with a protonated molecular mass [M+H]^+^ of 2959.7 Da. The analyses were performed using an AutoFlex II equipment (Bruker, Germany), applying 1 μL of HCCA matrix at 20 mg/mL.

The fractions that exhibited antimicrobial activity were analyzed by MALDI-TOF mass spectrometry. The fraction indicated in Figure 2 presented in its composition two components with distinct monoisotopic molecular masses. After the rechromatographic procedure (Figure 3A), the main component exhibited a protonated monoisotopic mass [M+H]^+^ of 3120.5 Da (Figure 3B), and the other component with a protonated monoisotopic molecular mass [M+H]^+^ of 2959.7 Da (Figure 3C).

### 3.2. Structural Characterization of Antimicrobial Peptides from B. raniceps

**Figure 4:**
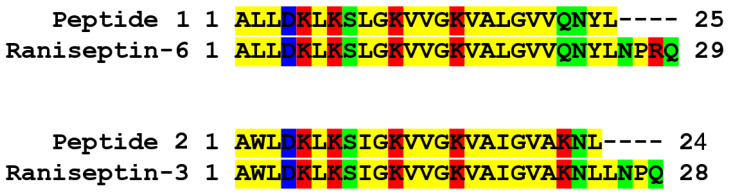
Alignment of peptides 1 and 2 (directly sequenced) isolated from *B. raniceps* skin secretion with the predicted amino acid sequences for the Raniseptins-3 and -6 identified in the *B. raniceps* cDNA library.

The native peptides were subjected to chemical sequencing by Edman degradation. The peptides found in fractions 1 and 2 (Figure 3A) resulted in the following two distinct partial sequences with 25 and 24 amino acid residues, respectively: Peptide 1 (^1^ALLDKLKSLGKVVGKVALGVVQNYL^25^) and Peptide 2 (^1^AWLDKLKSIGKVVGKVAIGVAKNL^24^). With these partial sequences, similarity searches were performed using the BLASTp tool and also the APD3 sequence bank. Peptide 1 corresponds to Raniseptin-6 (Rsp-6) and the peptide called 2 corresponds to Raniseptin-3 (Rsp-3), considering the molecular masses experimentally observed for these two peptides (Figure 3B,C), which allowed us to establish their complete primary structures (Figure 4).

These two Raniseptins were previously identified in a cDNA library constructed with *B. raniceps* skin [38]. Magalhães et al. described nine different Raniseptins, with five belonging to the A family with the N-terminal portion starting with AWL and four belonging to the B family with the N-terminal portion starting with ALL [38,39]. In the case of the Raniseptins isolated here, Rsp-3 belongs to family A and Rsp-6 belongs to family B.

Raniseptins-3 and -6 are cationic peptides (+4), hydrophobic and demonstrate GRAVY scores of 0.300 and 0.169, respectively (Table 1), features commonly found in AMPs [36,40].

**Figure 5:**
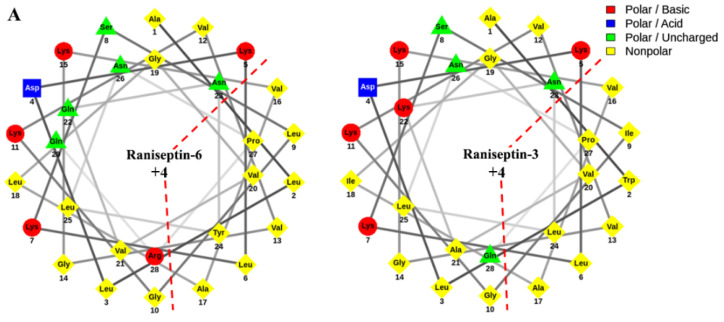

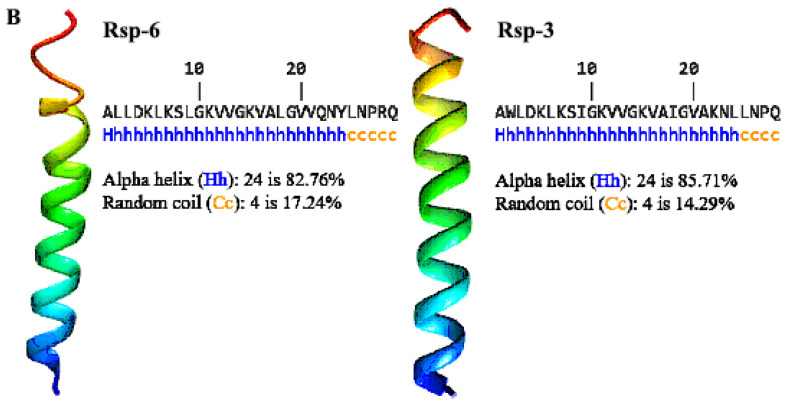
(**A**). Schiffer–Edmundson helical projections made using NetWheels of the antimicrobial peptides Raniseptins-3 and -6, highlighting the hydrophobic face of both peptides. (**B**). Secondary structure models for Raniseptins-3 and -6 using the I-TASSER server. In blue, the most electronegative region (N-terminal) can be observed, and in red, the electropositive region (C-terminal) can be observed.

The analysis of the helical wheel projections allowed us to observe that the antimicrobial peptides of *B. raniceps* tend to form amphipathic α-helices (Figure 5A). Secondary structure modelling revealed that both Raniseptins show tendencies to form α-helices with random N- and C-terminal regions (Figure 5B).

**Figure 6:**
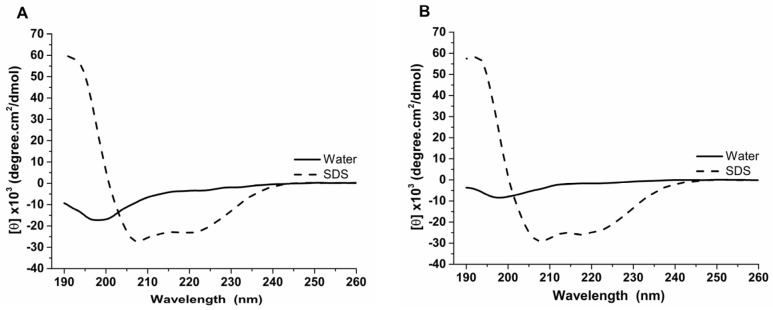
Circular dichroism analysis of the antimicrobial peptides of *B. raniceps* isolated in the present work. (**A**). Rsp-6 and (**B**). Rsp-3. Dichroic spectra were obtained from 190 to 260 nm, in aqueous solution and in the presence of SDS 35 mM. The thick black lines represent the dichroic curves of the samples in water, whereas the thin and dashed lines represent the dichroic curves in the presence of SDS 35 mM.

Our experimental analyses using circular dichroism revealed that the Raniseptins-3 and -6 adopt an α-helix conformation. The dichroic spectra of the peptides obtained in water at 25 °C showed mostly a negative dichroic band at 200 nm. These analyses revealed that both peptides presented disordered structures in aqueous solution; however, in the presence of SDS micelles, the peptides tended to structure themselves, mainly assuming a conformation in an α-helix (Figure 6). The dichroic spectra of the peptides in the presence of SDS 35 mM showed a significant shift of the negative dichroic band from 200 nm to the 208 nm and 222 nm regions, with [θ]_208nm_ values ranging between −16,000 and −31,000 degree·cm^2^/dmol, and a positive dichroic band at 190 nm. These results indicated that, in the presence of SDS, the peptides assumed a conformation predominantly in an α-helix.

### 3.3. Biological Characterization of Antimicrobial Peptides from B. raniceps

In order to advance in the biological characterization of the Raniseptins isolated in the present work, the peptides were produced using the Fmoc solid-phase peptide synthesis strategy. Mass spectrometry analyses were performed with the purified synthetic peptides to confirm the quality of the products produced by comparing the molecular masses obtained experimentally with the theoretical molecular masses expected for each of the peptides.

The synthetic antimicrobial peptides Rsp-3 and -6 were tested against four ATCC bacterial species, one multidrug-resistant *K*. *pneumonia* strain, and one ATCC yeast species, in addition to human erythrocytes, B16F10 murine melanoma cells, mouse fibroblasts NIH3T3 and MHV-3-infected L929.

Both peptides were shown to be very effective in inhibiting the growth of the Gram-negative bacteria *E. coli* and *K. pneumoniae*, with MIC values equal to 2 and 1 μM, respectively (Table 2), for both peptides. Inhibitory activity was slightly lower for the Gram-positive pathogens *S. aureus* and *S. epidermidis*, with Rsp-3 exhibiting MICs of 4 and 8 μM, respectively, for these bacteria (Table 2). The antimicrobial peptide Rsp-6 was also active against these microorganisms, showing MIC values equal to 32 and 8 μM for *S. aureus* and *S. epidermidis*, respectively (Table 2). Raniseptins were active against the resistant strain of *K. pneumoniae* carbapenemase (CAPB053), with MIC = 4 µM for both peptides.

These peptides were not able to completely inhibit the growth of the yeast *C. albicans*, even at the highest concentration used (128 μM) (Table 2). Even so, it was observed that both were able to markedly inhibit the growth of this yeast at this concentration (personal communications).

**Figure 7:**
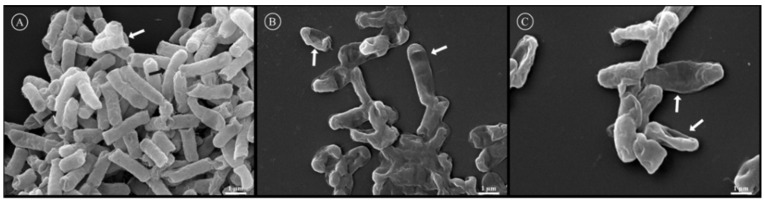
Analysis of the effect of Rsp-3 and -6 on the membrane of *Escherichia coli* (ATCC 25922) by SEM. (**A**) represents the control group incubated only with water, the white arrow shows the effect of the process of fixation and the black arrow indicates a bacterium with an intact membrane. (**B**,**C**) represent the effects induced by the presence of Rsp-6 and Rsp-3, respectively, on *E. coli* membranes, where the white arrows indicate the damages to the membranes by the peptides at the effective concentration of 2 µM.

The effect of Rsp-3 and -6 on the cell membrane of *E. coli* (ATCC 25922) was examined using Scanning Electron Microscopy (SEM). The grooves observed on the surface of the control group represent the effect of the fixation process and dehydration of the bacterial membrane (Figure 7A, indicated by a white arrow); however, it is possible to observe the presence of intact bacteria with a normal shape and smooth surface without any defects (Figure 7A, indicated by a black arrow). In the treated groups, various alterations in the cellular shape and surface of the bacteria were observed. The bacterial cells exhibited roughening surfaces and crimpled morphologies with pores on the cell surface (Figure 7B,C).

**Figure 8:**
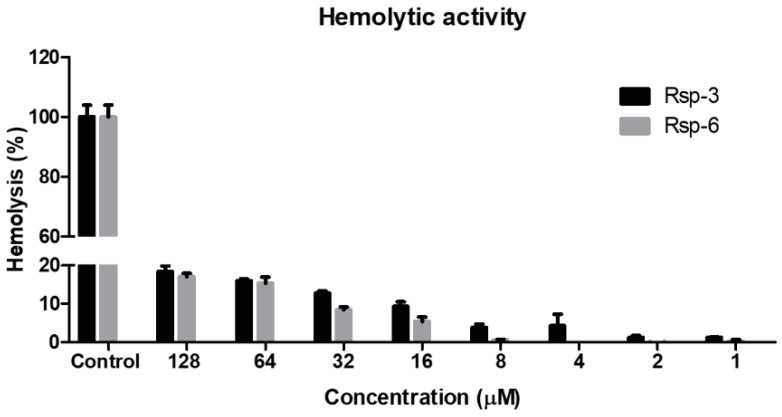
Hemolytic activity of Raniseptins-3 and -6 against human erythrocytes. Data points show mean ± SD.

The peptides exhibited dose-dependent hemolytic behavior and even at the highest concentration used (128 μM), the hemolytic activity detected was below 20%. Furthermore, at the concentration range in which the antimicrobial activity proved to be relevant (between 2 and 8 μM), hemolysis was below 5% (Figure 8).

**Figure 9:**
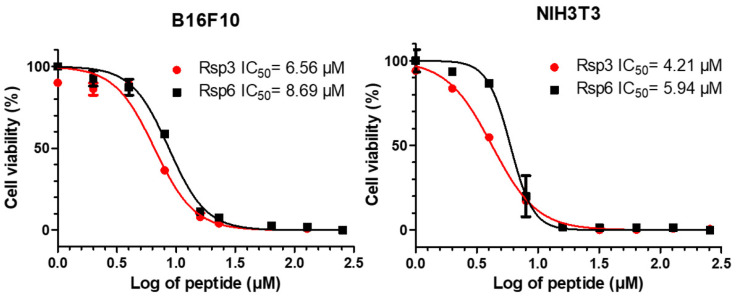
The antiproliferative effects of Raniseptins-3 and -6 on murine skin melanoma B16F10 cells and NIH3T3 mouse fibroblasts cells.

MTT-based assays for the measurement of antiproliferative activity for NIH3T3 murine fibroblast lines and B16F10 murine skin cancer cells were employed (Figure 9). The development of formazan crystals from the metabolism of MTT was detected after the cells were treated with the peptides. The Rsp-3 peptide showed an IC_50_ = 4.21 µM and IC_50_ = 6.56 µM for NIH3T3 and B16F10, respectively, whereas Rsp-6 showed an IC_50_ = 5.94 µM and IC_50_ = 8.69 µM for the same cells.

**Figure 10:**
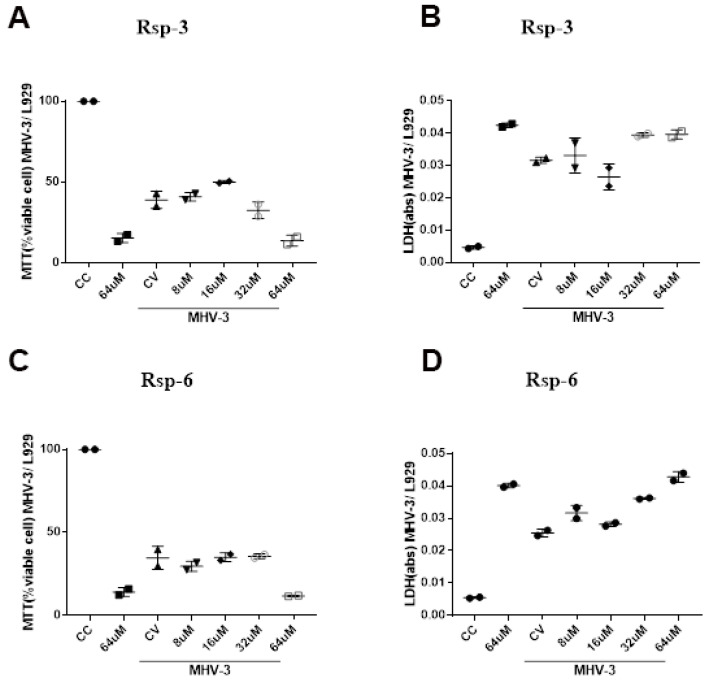
Biological effects of Raniseptins-3 and -6 on MHV-3-infected (L929) cells. Cell viability assessed by MTT assay ((**A**), Rsp-3 and (**C**), Rsp-6) and by LDH assay ((**B**), Rsp-3 and (**D**), Rsp-6).

LDH is an enzyme widely used for cytotoxicity evaluation, since it is released in the culture supernatant during cellular damage processes, and MTT is also a useful strategy used to assess the possible cell damage, since the tetrazolium salt of this compound is reduced to purple formazan crystals in the presence of mitochondrial dehydrogenases that are present in metabolically viable cells, serving as a good indicator of cell viability. Our results showed that the highest concentration of all the compounds tested exhibited high toxicity to the cell, which can be observed both in the MTT and LDH assay. In addition, MHV-3 infection was able to reduce cell viability after 16 h of infection; on the other hand, none of the tested compounds showed improvement trends in the MTT and LDH parameters of MHV-3 infected cells at the concentrations tested (Figure 10).

## 4. Discussion

The cutaneous secretion of amphibians includes a great variety of substances, and these compounds can be more or less expressed according to certain variables, such as food, the season of year (rainy or dry), cycle or phase of the individual’s development and even the animal collection location. For the genus *Boana*, few antimicrobial peptides have been isolated and characterized so far, and among them, Hylaseptin-P1 was found in the secretion of *B. punctata* [41], Hylin-a1 was isolated from *B. albopunctatus* [42], Cinerascetins was found in *B. cinerascens* [17] and Raniseptin-1 [38] and Figainins were isolated from the cutaneous secretion of *B. raniceps* [36,40].

The chromatographic profile of *B. raniceps* exhibited high complexity in its composition, presenting several chromatographic fractions, where the fractions of interest exhibited hydrophobic behavior after being eluted from 40% acetonitrile (Figure 2). In general, antimicrobial peptides are hydrophobic, with only a few exceptions [36,40,42]. The hydrophobicity of these molecules results from the presence of amino acids with apolar features, such as Leu, Ile, Val, Phe, Tyr and Trp. Its cationicity is related to the presence of the positively charged amino acids Lys and Arg [43]. When analyzing the primary structures of the peptides isolated in the present study, it is possible to observe that these peptides have a high percentage of hydrophobic and basic residues, justifying their physicochemical properties, such as high retention time, positive net charge and high hydrophobicity.

According to Kyte and Doolittle [44], it is possible to assess the degree of hydrophobicity by determining the mean hydropathicity index (GRAVY), which is calculated as the sum of the hydropathy values of all amino acids divided by the number of residues in the sequence, resulting in positive values for hydrophobic molecules. Raniseptins-3 and -6 exhibit GRAVY values of 0.3 and 0.169, respectively, indicating the hydrophobic profile of both peptides.

The peptides identified in the present study correspond to the Raniseptins-3 and -6 previously identified in a cDNA library produced from the skin of *B. raniceps* [38]. However, only Raniseptin-1 (Rsp-1) was isolated and characterized by Magalhães et al. [38], and due to the limited amount of material, its chemical synthesis was performed. Raniseptin-1 was significantly active against the same bacteria tested in the present work, with MIC values equal to 5 μM for *E. coli* and 20 μM for *S. aureus* [38].

Raniseptin-3 showed marginally higher antimicrobial activity against *E. coli* with MIC = 2 μM, but a strong effect on *S. aureus* with MIC = 4 μM in comparison to Rsp-1. Rsp-6 exhibited antimicrobial activity identical to that of Rsp-3 against the Gram-negative bacteria tested, although it was less effective against the Gram-positive bacterium *S. aureus*.

It is believed that the selectivity observed for AMPs may be related to the differences in the constitution of the membranes of Gram-negative and Gram-positive bacteria [36,45]. While the inner or cytoplasmic membranes of both groups of bacteria are similar, their outer envelopes are quite different. In Gram-positive bacteria, there is a layer of peptidoglycan (with associated teichoic acid) that allows the diffusion of AMPs through nano-sized pores. In Gram-negative bacteria, the peptidoglycan layer is thinner, and an additional external membrane formed mainly by LPS is found, which implies the need for AMPs to promote disturbance or rupture of the external and cytoplasmic membranes, resulting in a two-step process [46]. Rsp-3 is active against both types of bacteria, which can include interactions not only with lipopolysaccharides and lipoteichoic acid, but also with peptidoglycans.

Another factor that may influence the antimicrobial activity observed for the two Raniseptins could be associated with the fact that Rsp-3 has a larger hydrophobic face than Rsp-6, which may justify the more effective action against a wider spectrum of bacteria [47]. Cationicity does not seem to be a determining factor for the selectivity of these two peptides since they have the same net charge (+4). Some peptides described for the genus *Boana* are more effective against Gram-positive bacteria, such as Hylin a1 with a MIC value of 8 μM [42] and Cinerascetin 01 with a MIC equal to 10 μM against *S. aureus* [17]. Other peptides found in hylids also demonstrate greater action against Gram-positive bacteria, such as Brevinins with a MIC = 2.5–10 μM and Esculentins with a MIC = 2.5–5 μM [48].

The cytolytic effects of antimicrobial peptides are related to their cationic properties, with this effect being due to their interaction with the sialic acid molecules present on the surface of erythrocytes [49]. Sialic acid is negatively charged, which favors interaction with higher net charged peptides. In general, antimicrobial peptides have a net positive charge, ranging from +4 to +7, with a few exceptions represented by the peptides with a net negative charge, of which only 15 have been described in amphibian secretions [50]. The net positive charge of these molecules is directly related to the ratio between the basic amino acids, such as His, Lys and Arg, and acidic amino acids, such as Asp and Glu, present in these molecules [43]. In the case of Rsp-3 and -6 peptides, both have five positive residues in their sequences balanced by an Asp residue, resulting in a net charge equal to +4. Although cationicity is an important factor for their cytolytic activity against human erythrocytes, the peptides Rsp-3 and -6 demonstrated less than 20% hemolysis even at the highest concentration used of 128 μM, in contrast to what was observed for Figainin 2 and Hylin a1, which showed HC_50_ values equal to 48.9 and 18.6 μM, respectively [36,42].

Both peptides were also tested against mouse cell lines and exhibited antiproliferative and cytotoxic effects on murine melanoma B16F10 cells and on L929 and NIH3T3 mouse fibroblasts. The observed results may be related to the cationic properties of the peptides studied [49], which lead to their stronger interaction with anionic molecules such as phosphatidylserine, O-glycosylated mucins, sialylated gangliosides and heparin sulfate present in cancer cell membranes that may or may not be overexpressed [51,52]. Other hydrophobic properties can also affect the cytotoxicity process.

For comparitive purposes, the peptide Dermaseptin-L1, identified in the secretion of *Agalychnis lemur* (Hylidae: Phyllomedusinae), exhibits stronger selectivity over cancerous cell lines than normal cells. For HepG2 human hepatoma-derived cells, the peptide Dermaseptin-L1 showed a LC_50_ = 45 µM; nevertheless, for human erythrocytes, this peptide exhibited a LC_50_ = 200 µM [53]. The Figainin 1 and 2 peptides, which belong to the same family, are present in the cutaneous secretion of the species *B. raniceps*. Figainin 1 peptide has demonstrated activity against the human cervical adenocarcinoma HeLa, human mammary adenocarcinoma cell line MCF-7, and the murine skin melanoma cell line B16F10, with IC_50_ values equal to 11.1, 13.7, 10.5 µM, respectively. For the Figainin 2 peptide, an IC_50_ = 12.8 µM was observed for B16F10 and IC_50_ = 15.3 µM for MCF-7; however, these two peptides demonstrate potent hemolytic action against human erythrocytes [36,40].

Currently, the use of antimicrobial peptides as potential drugs against emerging infectious viral pathogens, such as dengue virus, Zika virus, and SARS-CoV-2, is under investigation [54,55] and in the present study, we tested Raniseptins-3 and -6 against the MHV-3 virus, a murine hepatitis virus that shares the same genus (*Betacoronavirus*) with the SARS-CoV-2 virus, the severe acute respiratory syndrome coronavirus-2 that infected millions of people worldwide [56,57,58]. MHV is a well-studied animal coronavirus that serves as a safe and cost-effective model used to investigate infections by coronaviruses [59]. Before testing the compounds of interest on a SARS-CoV-2 antiviral assay, the MHV model can be initially used and, if deleterious effects on this virus are detected, the effects on SARS-CoV-2 can be evaluated. Unfortunately, the Raniseptins peptides were highly toxic for the host L929 cells.

As an alternative, the adverse effects observed on mammalian cells can be avoided with the use of nanoencapsulation and delivery strategies [60,61].

## 5. Conclusions

In conclusion, the antimicrobial peptides isolated from the cutaneous secretion of *Boana raniceps* exhibited therapeutically interesting antimicrobial properties, which may contribute to the development of new antimicrobial agents in order to combat the current crisis in the treatment of infections caused by resistant microorganisms, reinforcing the need to advance in the exploration, for pharmaceutical purposes, of the Brazilian anuran’s biodiversity.

## Figures and Tables

**Table 1:** Physicochemical properties of Raniseptins-3 and -6.

Peptide	Mass calc. (Da)	Mass obs. (Da)	Net Charge	Hydrophobic Face	Hydrophobicity <H>	GRAVY
Raniseptin-3	2958.77	2958.7	+4	VIPWVVLLA	43.77	0.300
Raniseptin-6	3119.85	3119.5	+4	VLPLVVLYA	43.60	0.169

**Table 2:** Antimicrobial activities of Raniseptins-3 and -6 (MIC in μM).

Microorganisms	Rsp-3	Rsp-6
Gram-negative bacteria		
*E. coli* (ATCC 25922)	2	2
*K. pneumoniae* (ATCC 13883)	1	1
*K. pneumoniae* carbapanemase (KPC CAPB053)	4	4
Gram-positive bacteria		
*S. aureus* (ATCC 25923)	4	32
*S. epidermidis* (ATCC 12228)	8	8
Yeast		
*C. albicans* (ATCC 14053)	>128	>128

## Data Availability

The authors confirm that the data supporting the findings of this study are available within the article.
